# Temporal phenotypic variation of spinach root traits and its relation to shoot performance

**DOI:** 10.1038/s41598-024-53798-3

**Published:** 2024-02-08

**Authors:** Ji Liu, Jiapeng Shui, Chenxi Xu, Xiaofeng Cai, Quanhua Wang, Xiaoli Wang

**Affiliations:** Development and Collaborative Innovation Center of Plant Germplasm Resources, College of Life Sciences, Shanghai Normal University, Shanghai, 200234 China

**Keywords:** Physiology, Plant sciences

## Abstract

The root system is important for the growth and development of spinach. To reveal the temporal variability of the spinach root system, root traits of 40 spinach accessions were measured at three imaging times (20, 30, and 43 days after transplanting) in this study using a non-destructive and non-invasive root analysis system. Results showed that five root traits were reliably measured by this system (RootViz FS), and two of which were highly correlated with manually measured traits. Root traits had higher variations than shoot traits among spinach accessions, and the trait of mean growth rate of total root length had the largest coefficients of variation across the three imaging times. During the early stage, only tap root length was weakly correlated with shoot traits (plant height, leaf width, and object area (equivalent to plant surface area)), whereas in the third imaging, root fresh weight, total root length, and root area were strongly correlated with shoot biomass-related traits. Five root traits (total root length, tap root length, total root area, root tissue density, and maximal root width) showed high variations with coefficients of variation values (CV ≥ 0.3, except maximal root width) and high heritability (H^2^ > 0.6) among the three stages. The 40 spinach accessions were classified into five subgroups with different growth dynamics of the primary and lateral roots by cluster analysis. Our results demonstrated the potential of in-situ phenotyping to assess dynamic root growth in spinach and provide new perspectives for biomass breeding based on root system ideotypes.

## Introduction

Root is a major organ for nutrient and water uptake and plays an important role in plant growth, development and adaptation to different edaphic environments^1–4^. With the development of root phenotyping, there has been an increase in root system investigations. Large variation in the root phenotypes has been found in diverse crop accessions such as barley^5,6^, soybean^7,8^, rapeseed (*Brassica napus* L.)^9^, maize^10^, wheat^11^, rice^12^, watermelon^13^, and *Phaseolus vulgaris*^14^, exhibiting the genetic potential of various genotypes to be utilized by plant breeders. Some root traits have been exploited to improve nutrient use efficiency, yield, and abiotic stress adaptation. For example, a root ideotype with deeper and steeper roots is benefit for N acquisition^15,16^, while a deep and vigorous root system is often associated with high yields in cereal crops^17,18^. In maize, primary root depth was positively correlated with salt tolerance during early growth^19^, and low crown root number was suggested as a selection criterion for screening maize with low nitrogen tolerance^20^. Root number and fine root growth are critical for identifying drought adaptability in cassava^21^ and oats^22^, respectively.

The root characteristics and development are also particularly essential for spinach (*Spinacia oleracea* L.). Spinach is an important leafy vegetable cultivated all over the world. It has a shallow root system and a high nitrogen requirement during the vegetative growth stage. With the advancement of protected horticultural technology and increased market demand for vegetable supply, spinach cultivation methods are becoming increasingly diverse, ranging from traditional open field cultivation to soilless indoor farming. The changes of root environment impose more demands on the adaptation of new cultivars to cope with various environmental, nutritional or, ionic stresses^23–25^. Therefore, understanding the structure and development of spinach root, as well as the genetic diversities among root traits could help identify optimal genotypes desirable for spinach cultivar improvement. However, due to the fragility and complexity of spinach root systems, selection and breeding work in spinach has mainly focused on shoot traits, but only limited work is available on spinach root^27–30.^ With the exception of a study by Awika et al*.*^24^ which used the supervised machine learning algorithm to predict several important root traits, almost all existing studies on spinach roots focus only on root biomass and measured them by manual^26,31^. At present, the non-destructive image acquisition of spinach root system has not been reported.

In particular, the architecture of the root system depends on continuous root growth in a given space and time^32–34^, while most root phenotypic studies in spinach only focus on the final traits at the tested stage, but ignore the differences in root characteristics between different developmental stages^24,26^. It is therefore necessary to investigate the temporal variability of the root system during spinach growth and its impact on aboveground performance.

The X-ray imaging system as a well-established technique for non-invasive imaging has been designed and built for root visualization studies for more than 20 years^35–38^. It can directly image the plant root system at different growth periods without damaging the root system, which is suitable for repeatedly tracking plant root growth over a long period of time. Moran et al.^39^ used X-ray absorption and phase contrast imaging to study the distribution of plant roots in soil. Pierret et al*.*^40^ used a 60 kV, 0.33 mA X-ray source to obtain a in-situ and high-resolution two-dimensional X-ray images of the roots of a narrow leafed lupin. Gregory et al*.*^41^ effectively reconstructed the three-dimensional root images of wheat and rape using a X-ray micro-tomography imaging system. Using the similar technique, Kodrzycki et al.^42^ compared the poplar clones roots grown in soil or foam substrates by a commercial system RootViz FS (Plant Root X-ray Scanning Imaging System), and found that the overall rank of clonal performances were consistent regardless of rooting medium, suggesting that the technique is effective at detecting roots and monitoring root growth. However, whether this non-invasive X-ray scanning imaging technique is suitable for the detection of spinach root remains uncertain.

Therefore, the purpose of this study was: (1) to test the effectiveness of non-destructive analysis of spinach root traits, and (2) to characterize the temporal dynamics of root traits and determine their relationship with shoot characteristics. For this purpose, phenotypic variability in several root and shoot traits among 40 spinach accessions was measured at three times throughout the vegetative growth in this study. We hypothesized that the root characteristics of spinach germplasm exhibit significant temporal variation and that these dynamic variations may affect the shoot performance. These results will provide new insights into the dynamic diversity of root development in spinach and help to select representative accessions with contrasting root characteristics for further study.

## Materials and methods

### Plant materials and growth in RootViz FS system

A total of 40 cultivated spinach accessions from 15 countries with good representativeness and genetic diversity that were recently reported by Cai et al*.*^28^ were used in this study. The experimental research on plants, including the collection of plant material, complied with relevant institutional, national, and international guidelines and legislation. The details of these accessions are shown in Supplementary Table S1.

**Figure 1:**
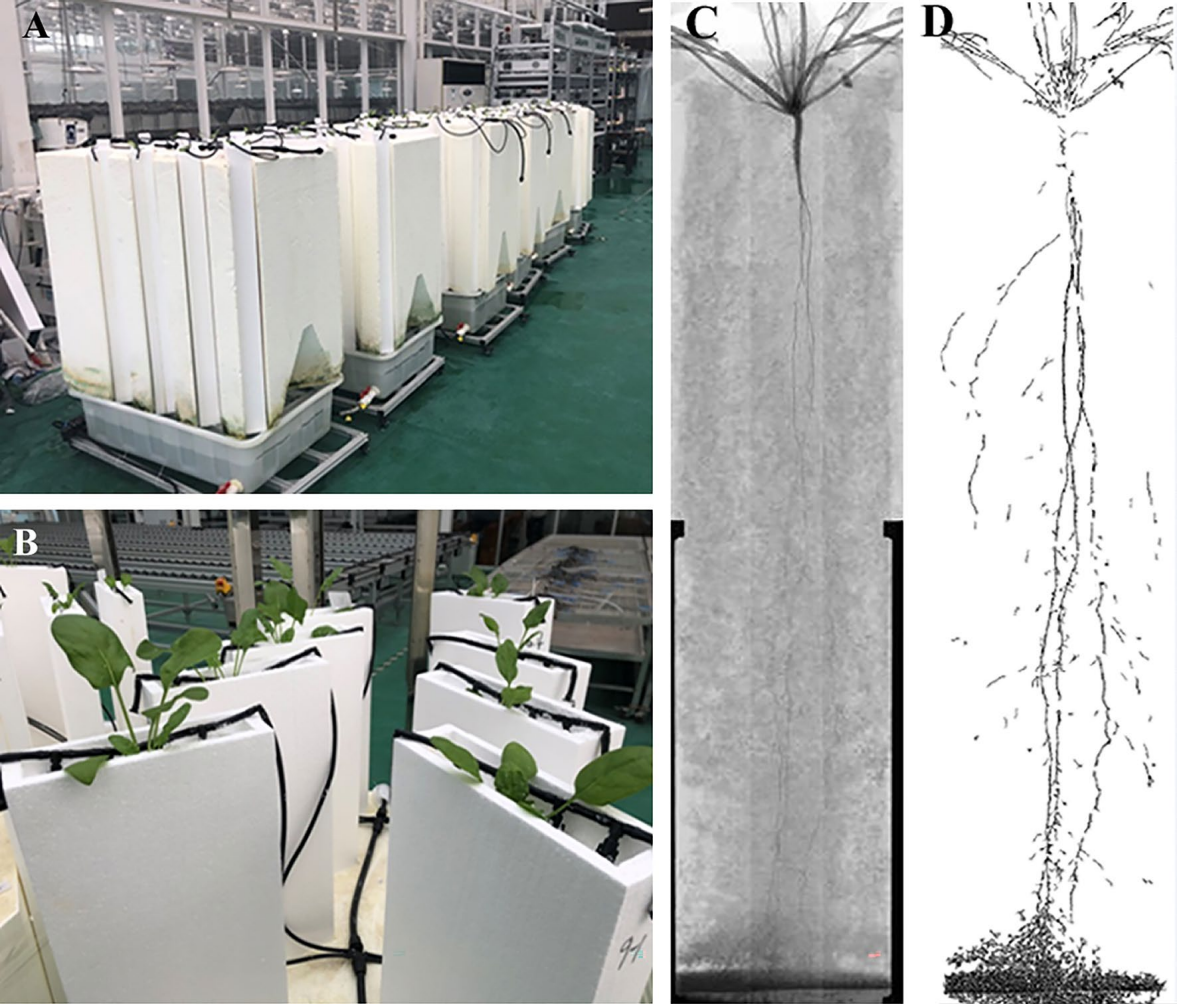
The rhizoboxes of the RootViz FS system in a greenhouse (**A**), a close shot of plants in the rhizoboxes (**B**), and an example of plant roots scanned by X-ray computed tomography without (**C**) and with filters (**D**).

Spinach seeds were surface sterilized with 10% bleach and 70% ethanol, and then incubated at 4 °C for 24 h for pre-germination. After that, the seeds were sown in trays filled with polystyrene foam beads for germination in a 25 °C growth chamber under 1/4 Hoagland solution. Two weeks after sowing, the uniformly and healthy germinated seedlings (root length was about 10 cm) were transplanted into acrylic rhizoboxes (25 × 4 × 100 cm) for further cultivation (Fig. 1). Each rhizobox was filled with polystyrene foam beads (1–3 mm) as culture mediums, and one plant was planted. There were 160 rhizoboxes in total, with four replicates for each accession (three replicates with consistent growth were selected for subsequent analyses). Each rhizobox was supplied with 1/2 Hoagland solution by an automated irrigation system. The irrigation system included a reservoir, a water pump, an irrigation pipe system, and a timer. The timer is used to control the supply of nutrient solution, with a liquid supply of 5 min and a stop of 2 min.

The pH was frequently monitored, and the solutions in reservoirs were renewed weekly. The experiment was conducted in a greenhouse with an average temperature of about 20 °C/10 °C (day/night) during the experimental period. Plants were harvested at 43 days after transplanting (DAT), when most of the roots were beginning to reach the bottom of the rhizoboxes. At harvest, plant morphology data and biomass were collected.

### Plant image scanning

For phenotypic variation analysis, the root morphological traits were measured with the RootViz FS system (Phenotype Screening Corporation, USA) at three times (20, 30, and 43 DAT). Root images were scanned in situ by the X-ray radiography. The X-ray source was operated at 90 kV, 1.5 mA, and the beam filtered with 20 μm of palladium and give a quasi-monoenergetic spectrum centered about the 25 keV silver peak. After X-ray transmission imaging, a 16-bit scan root image with a resolution of 2940 × 2304 pixels per inch was obtained and then organized into RhizoTraits Softway, an image analysis component of the RootViz FS system for analysis. After proper filtering (40% resolution), interpolation, and encapsulation preprocessing, five valid root traits (total root length, tap root length, total root area, root tissue density, and maximal root width) were directly generated by RhizoTraits.

Nine shoot traits (plant height, plant breadth, leaf width, leaf length, petiole width, petiole length, leaf shape, shape of leaf apex, and shoot fresh weight) were measured manually on the same day as root image acquisition. Meanwhile, a commercial phenotyping system, Scanalyzer 3D (LemnaTec GmbH, Germany) was also used for shoot image acquisition from the top of these plants. The image processing procedures of Scanalyzer 3D were described elsewhere^43,44^. Two values related to shoot biomass, object area (OA) and object volume (OV, calculated by multiplying the OA by the height of the plant height) were generated from this system. After the final photographing, the roots were cut from the shoots, and the fresh weight of roots and shoots were measured separately. Hand measurements were also recorded for tap root length and maximal root width. The descriptions of all traits are listed in Table 1.

**Table 1:** Description of root and shoot-related traits in 40 spinach genotypes characterized in the RootViz FS system and the Scanalyzer 3D phenotyping system.

Tissue	Trait	Abbreviation	Description	Unit
Shoot	Plant height	PH	shoot height	cm
	Plant breadth	PB	the widest point of plant canopy	cm
	Leaf length	LL	the longest length of the leaf blades from base to tip	cm
	Leaf width	LW	the maximum width of the leaf blades	cm
	Petiole length	PL	the longest length of the petioles from base to tip	cm
	Petiole width	PW	the diameter of the thickest part of the petioles	cm
	Leaf shape	LS	the shape of the middle-canopy leaves	nearly orbicular = 1, ovate = 2, elliptic = 3, halberd = 4,
	Shape of leaf apex	SLA	shape of the tip of the middle-canopy leaves	acute = 1, subacute = 2, rounded = 3
	Shoot fresh weight	SFW	total shoot fresh mass per plant	g
	Object area	OA	plant surface area calculated by the number of object pixels	cm^2^
	Object volume	OV	object area multiply plant height	dm^3^
Root	Total root length	RL	total length of all roots per plant	m
	Tap root length	TRL	distance from root base to tip	cm
	Total root area	RA	root surface area	cm^2^
	Root tissue density	RTD	total root length per rhizobox area	m/m^2^
	Maximal root width	MRW	the maximal extent of the root system in horizontal direction	mm
	Mean growth rate of total root length	MRG_RL	change in total tap root length per day	m/d
	Root fresh weight	RFW	total fresh mass of all roots per plant	g
	Mean growth rate of tap root	MRG_TRL	change in total tap root length per day	cm/d
	Specific root length	SRL	total root length per unit root fresh mass	m/g

### Data analysis

The mean growth rate of total root length (MRG_RL) and the mean growth rate of tap root (MRG_TRL) were calculated using the following equation:

$$MRG=\frac{L-{L}_{0}}{D-{D}_{0}}$$

In the above formula, *MRG* is MRG_RL or MRG_TRL (m/d or cm/d); *L* is the RL or TRL at the later imaging period; *L*_*0*_ is the RL or TRL at the early imaging period; D is the number of DAT at the later imaging period; *D*_*0*_ is the number of DAT at the early imaging period.

The phenotypic data were analyzed for descriptive statistics, significant differences (One-way ANOVA, least significant difference test), and broad-sense heritability (h^2^) using SPSS Statistics 25 (IBM, USA). The broad-sense heritability (h^2^) of root traits was determined using the equation: h^2^ = V_g_/(V_g_ + V_gt_/nReps + V_e_/nReps/nT), where V_g_ and V_e_ is the genotype and the residual error variance components, respectively; V_gt_ is the variance component of genotype by imaging time interaction; nReps and nT are the number of replicates and imaging times, respectively. Correlations between root and/or shoot traits were calculated using Spearman’s correlation coefficient using SigmaPlot 12.5 (Systat Software, USA). A simple linear regression analysis was applied to further verify their relationship. ANOVA was used to determine the relationship between the root and leaf shape traits in SPSS Statistics 25. Log (base 2) transformed ratios were used for hierarchical clustering analysis using SPSS Statistics 25 (IBM, USA) with the average-linkage method, and squared Euclidean distance was selected to establish clusters. The heatmap was visualized with R version 3.5.2 (R Foundation for Statistical Computing, Austria). To compare the root traits in each group, the normalized values for each trait were calculated as the quotient of the trait value in one group minus the mean value of all groups divided by the mean value.

The overall root performance of spinach germplasm was evaluated using a fuzzy comprehensive evaluation method of the membership function value (MFV). The root MF value was calculated using the following equation^45^: ${x}_{i}=\frac{x-{x}_{min}}{{x}_{max}-{x}_{min}}$, where *X*_*i*_ represents the MFV of the *i*th germplasm accession in a given trait, *X* is the value of a given germplasm accession for the given parameter, and *X*_*max*_ and *X*_*min*_ are the maximum and minimum values of the given trait across the germplasm accessions, respectively. Finally, the mean MFV across traits was computed to rank the germplasm accessions.

## Results

### Comparison of RootViz FS and manual measurement

After scanning, many indicators were obtained by the RootViz FS system, such as root surface area (RA), total root length (RL), number of intercepts at different depths for different sizes of roots, area of intercepts at different depths for different sizes of roots, total number of intercepts at different depths for different sizes of roots, and diameter of intercepts at different depths for different sizes of roots. However, considering the reliability and/or accuracy and continuity under three times measurements, only five root traits (RA, RL, tap root length, root tissue density, and maximal root width) were left for further analysis.

To validate the reliability of RootViz FS system for spinach root measurement, the tap root length (TRL) and maximal root width (MRW) were measured after harvest by manual. Although some differences were found, linear regression analysis showed that both TRL (r^2^ = 0.816) and MRW (r^2^ = 0.743) measured by RootViz FS correlated strongly with their manually measured analogs, indicating the suitability of this method to evaluate this traits in spinach (Figure S1).

### Variation in root and shoot traits

Descriptive statistics and ANOVA analysis showed that all measured root traits differed significantly among accessions across the three imaging days (*P* < 0.05) (Table 2). Most traits (7 of 10) had high variations with coefficients of variation values (CV ≥ 0.3) among the three periods. The mean growth rate of total root length (MGR_RL), specific root length (SRL), root fresh weight (RFW), and mean growth rates of tap root length between 20 and 30 DAT (MRG_TRL12) had a relatively larger variation among accessions (CVs ≥ 0.5). Total root length (RL), root surface area (RA), and root tissue density (RTD) varied more than fivefold among the accessions across the three imaging times. However, two traits, MRW and TRL had relatively smaller variations, whose CVs were less than 0.3. The CVs of RL, RA, and RTD generally increased with the prolonging of cultivation, while the CVs of MRW, MRG_RL, and MRG_TRL showed opposing patterns.

**Table 2:** Descriptive statistics and heritability of root traits in 40 spinach accessions characterized in the RootViz FS system at 20 (1st), 30 (2nd), and 43 (3rd) days after transplanting (DAT).

Root trait	Abbreviation	Minimum	Maximum	Mean	Fold	Standard deviation	CV	Significance	H^2^
RL	RL_1	0.83	5.60	2.59	6.77	1.06	0.41	0.00	0.78
	RL_2	1.38	9.20	3.88	6.67	1.56	0.40	0.00	
	RL_3	3.69	27.50	10.03	7.45	4.70	0.47	0.00	
RA	RA_1	2.47	16.52	8.33	6.70	3.54	0.42	0.00	0.79
	RA_2	4.07	25.54	11.71	6.28	4.62	0.39	0.00	
	RA__3	11.87	66.89	32.64	5.64	12.98	0.40	0.00	
RTD	RTD_1	106.69	773.56	401.68	7.25	146.53	0.36	0.00	0.78
	RTD_2	194.68	1299.99	575.40	6.68	218.22	0.38	0.00	
	RTD_3	635.58	3662.62	1464.42	5.76	664.21	0.45	0.00	
MRW	MRW_1	21.11	134.20	93.27	6.36	25.56	0.27	0.00	0.75
	MRW_2	62.87	156.61	121.95	2.49	19.22	0.16	0.00	
	MRW_3	107.80	178.78	147.70	1.66	19.04	0.13	0.00	
TRL	TRL_1	21.11	58.67	37.76	2.78	7.90	0.21	0.00	0.69
	TRL_2	29.56	77.11	50.73	2.61	10.83	0.21	0.00	
	TRL_3	53.62	98.99	87.18	1.85	10.02	0.17	0.00	
MRG_RL	MRG_RL12	0.00	0.38	0.11	321.72	0.11	1.04	0.00	ns
	MRG_RL23	0.00	0.70	0.18	374.64	0.18	0.96	0.00	
	MRG_RL13	0.06	0.83	0.24	14.40	0.16	0.65	0.00	
MRG_TRL	MRG_TRL12	0.31	4.61	1.62	15.09	0.84	0.52	0.00	ns
	MRG_TRL23	0.36	2.53	1.58	7.00	0.47	0.30	0.00	
	MRG_TRL13	0.73	2.19	1.59	2.99	0.30	0.19	0.01	
Root fresh weight/g	RFW_3	1.60	38.28	12.65	23.93	8.31	0.66	0.00	ns
Specific root length/(m/g)	SRL_3	0.30	3.28	1.03	11.09	0.65	0.63	0.00	ns
Root-shoot ratio	RS_3	0.10	0.50	0.28	5.00	0.10	0.37	0.00	ns

The number after the underscore indicates the order of image acquisition.

MRG_RL12, mean growth rate of total root length between the 1st and 2nd sampling periods; MRG_RL23, mean growth rate of total root length between the 2nd and 3rd sampling periods; MRG_RL13, mean growth rate of total root length between the 1st and 3rd sampling periods; MRG_TRL12, mean growth rate of tap root length between the 1st and 2nd sampling periods; MRG_TRL23, mean growth rate of tap root length between the 2nd and 3rd sampling periods; MRG_TRL13, mean growth rate of tap root length between the 1st and 3rd sampling periods.

The high heritability (H^2^) of five root traits (RL, RA, RTD, MRW, and TRL) was found across the three imaging times, though varying degrees of existed among traits (Table 2 and Supplementary Table S2). RA showed the largest heritability (0.79) than others, with the TRL being the least heritable (0.69).

Variation in shoot traits at three image times, including object area (OA) and object volume (OV), was also relatively high among accessions (CV > 0.4). Plant height (PH), petiole length (PL), leaf width (LW), and shoot fresh weight (SFW) at 43 DAT had a relatively larger variation (CV > 0.3) than those at 20 and 30 DAT. Petiole breadth (PB), leaf length (LL), and petiole width (PW) among the three stages showed smaller divergence with CV values lower than 0.4 (Supplementary Table S3). Compared to the early period, more considerable variations in shoot traits were found at the later stage.

### Correlation among phenotypic traits in RootViz FS system

Spearman’s correlation analysis showed a strong correlation between the same root traits across the imaging stages, such as RA, RTD, and TRL. RL and MRW at the early vegetative stage only correlated with those at the middle stage (Table 3). Strong correlations between the same shoot traits were also observed among different developmental stages, and their correlations were much stronger than those of root traits, suggesting a greater diversity of dynamic growth patterns in roots than shoots.

**Table 3:** The Spearman’s correlations of the measured shoot and root traits between the two imaging stages.

	20 DAT/30 DAT	30 DAT/43 DAT	20 DAT/43 DAT
RL	0.67**	0.29	0.08
RA	0.66**	0.33*	0.05
RTD	0.65**	0.44**	0.2
TRL	0.83**	0.46**	0.47**
MRW	0.46**	0.16	0.09
PB	0.87**	0.82**	0.85**
PH	0.77**	0.79**	0.66**
LL	0.79**	0.69**	0.76**
LW	0.75**	0.62**	0.46**
PL	0.87**	0.89**	0.84**
PW	0.55**	0.43**	0.46**
OA	0.93**	0.80**	0.68**
OV	0.92**	0.81**	0.69**

*RL* Total root length, *RA* Total root area, *RTD* Root tissue density, *TRL* Tap root length, *MRW* Maximal root width, *PB* Plant breadth, *PH* Plant height, *LL* Leaf length, *LW* Leaf width, *PL* Petiole length, *PW* Petiole width, *OA* Object area, *OV* Object volume, *DAT* Days after transplanting

*significance at *P* < 0.05, **significance at *P* < 0.01.

Regardless of the examined periods, most of the selected root traits, such as RL, RA, and RTD, significantly correlated with each other (except TRL and MRW, Supplementary Table S4). However, TRL strongly and positively correlated with RA at both the 30 DAT and 43 DAT but not at the 20 DAT, while TRL correlated positively with RTD only at the final imaging (43 DAT). TRL did not correlate with MRW at all stages. At 43 DAT, SRL had a weak positive correlation with PH and PL, but a negative correlation with RL, RA, and RTD. The RA, RL, and RTD were positively correlated with RFW, indicating these traits can be used for root biomass prediction.

**Figure 2:**
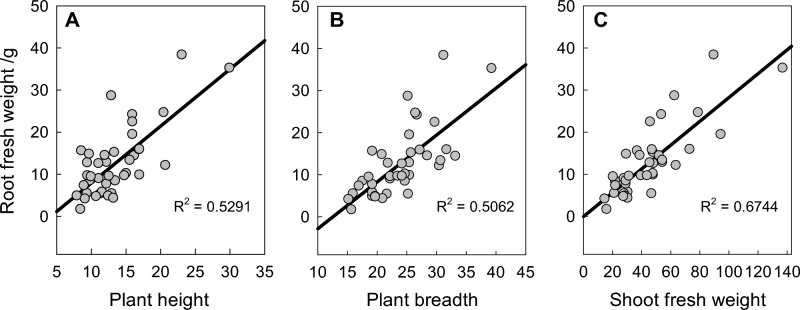
Linear regressions between root fresh weight and (**A**) plant height, (**B**) plant breadth, and (**C**) shoot fresh weight of 40 spinach accessions grown in the RootViz FS system for 43 DAT.

Unlike the root traits, all the shoot traits correlated with each other among the three stages. Among them, the OV at 43 DAT has the highest correlation with SFW, which could serve as proxies for shoot biomass at the two first imaging. As expected, significant correlations were found between root and shoot traits, but great differences existed among different imaging periods (Supplementary Table S4). At 20 DAT, a weak correlation was found between TRL and four shoot traits, PH, LW, OA, and OV, while other root traits showed no significant correlation with shoot traits. At 30 DAT, RL, RA, and TRL were weakly correlated with OA and OV, and TRL was also weakly correlated with PH and LL. Different from the first and second stages, at 43 DAT, most of the root biomass-related traits, RFW, RL, RA, and RTD, were highly correlated with shoot fresh weight (SFW) and other shoot traits, such as plant height (PH), plant breadth (PB), and OV. TRL also correlated with LL, PW, OA and OV, but no correlation was found between TRL and SFW. Regression analysis also indicated a significant correlation between PH, PB, SFW, and RFW at 43 DAT (Fig. 2). The results showed that spinach shoot traits correlate with TRL at early growth stages, but more root traits (RL, RA, and RTD) may have a higher correlation with shoot traits at a later period.

### Root traits and leaf shape

Not only the quantitative morphological traits, but the two shoot qualitative traits, leaf shape and shape of leaf apex, were also related to root traits. Take the results at 43 DAT as an example, the accessions with nearly orbicular leaf shapes had the lowest RA, RTD, TRL, MRG_TRL, RL, MRG_RL and RFW among the four types of leaf shapes (Table 4). Similarly, the spinach plants with acute leaf apex had significantly greater RA, RTD, MRG_RL, and RFW than those with rounded leaf tips, but no difference in RL, TRL, or MRG_TRL was found between the accessions with the three shapes of leaf apex. Considering the leaf apexes of hastate leaves are mostly acute, while the leaf apexes of nearly orbicular leaves are mostly rounded, we speculated that spinach with halberd-shaped leaves may have larger root systems than those with nearly orbicular-shaped leaves.

**Table 4:** Seven root traits and shoot fresh weight in spinach accessions with different leaf shapes and shapes of leaf apex at 43 days after transplanting (DAT).

Characters	RL	RA	RTD	MRG_TRL	RFW	TRL	MRG_RL	SFW
Leaf shape								
1: nearly orbicular	6.37 b	18.06 b	758.61 b	0.02 b	4.08 c	34.890 b	0.03 b	36.02 c
2: ovate	9.85 a	29.79 a	1357.95 a	0.04 a	8.64 b	48.18 a	0.04 a	39.92 bc
3: elliptic	8.75 a	28.79 a	1226.94 a	0.06 a	9.82 b	50.54 a	0.04 a	42.67 b
4: halberd	12.41 a	36.88 a	1675.7 a	0.08 a	16.76 a	53.12 a	0.05 a	50.89 a
Shape of leaf apex								
1: acute	11.85 a	37.29 a	1642.19 a	0.09 a	18.51 a	52.02 a	0.05 a	43.20 a
2: subacute	10.49 a	31.64 ab	1456.63 ab	0.06 ab	10.17 b	50.48 a	0.04 a	44.60 a
3: rounded	7.80 a	23.80 b	999.39 b	0.02 b	6.66 b	48.18 a	0.04 a	39.33 b

Different letters meant a significant difference among leaf shape or shape of leaf apex (*P* < 0.05).

*RL* Total root length, *RA* Total root area, *RTD* Root tissue density, *TRL* Tap root length, *MRW* Maximal root width, *MRG_TRL* Mean growth rate of tap root length, *MRG_RL* Mean growth rate of total root length, *SFW* Shoot fresh weight.

### Cluster analysis

**Figure 3:**
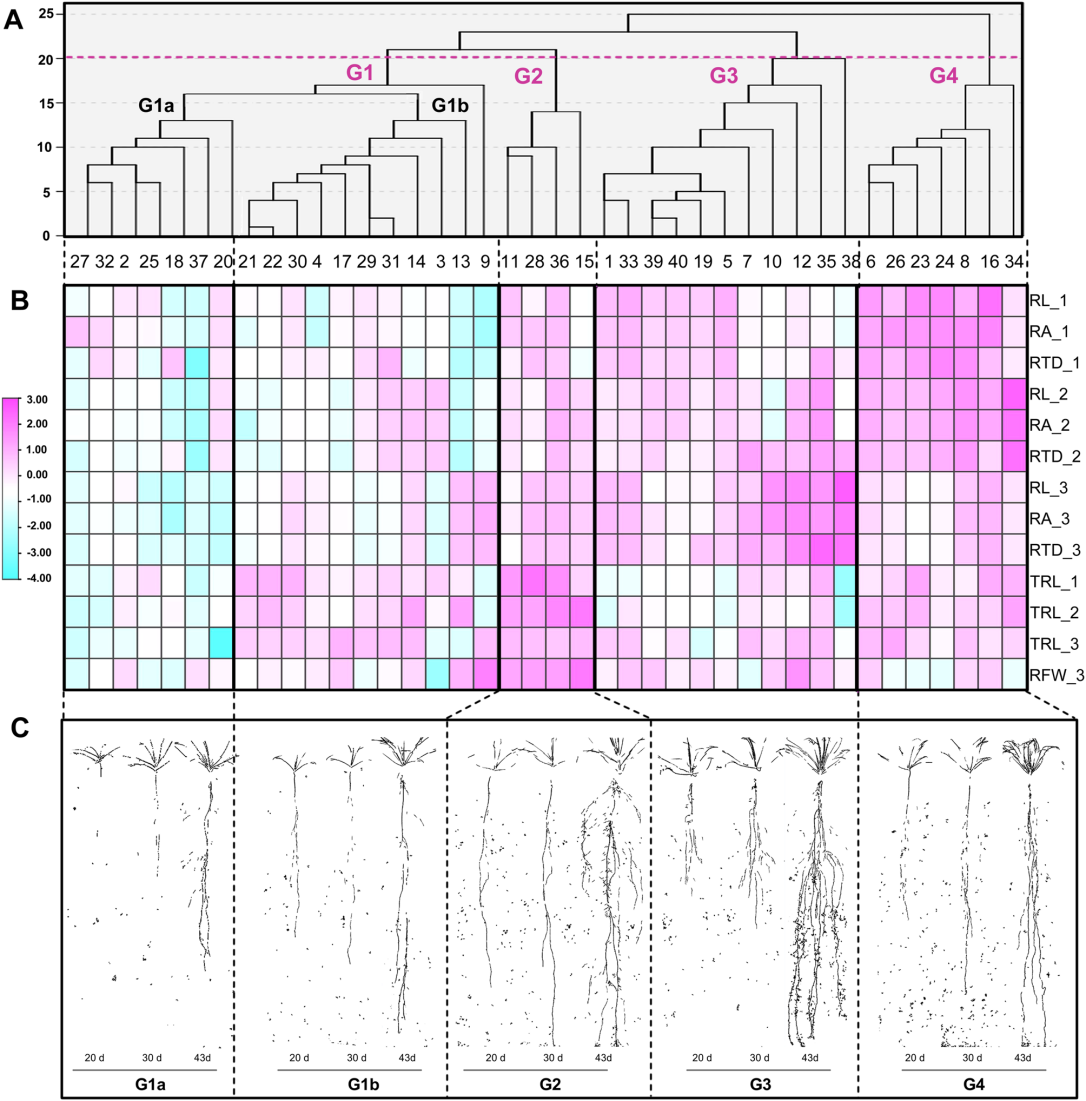
Dendrogram (**A**), heatmap of hierarchical clustering on five selected root traits of 40 spinach accessions at three time points (**B**), and sample root X-ray images of accessions (accessions from left to right were No. 37, No. 21, No. 36, No. 35, and No. 24, respectively) under the five identified subgroups (**C**). The root traits of each accession have been normalized using the Log (base 2) transformation. Hierarchical clustering of accessions was performed using the average-linkage method and visualized within R software. All accessions were classified into four groups: Group 1 (G1), Group 2 (G2), Group 3 (G3), and Group 4 (G4) at a squared Euclidean distance of 20, and Group 1 was further divided into two subgroups: Group 1a (G1a) and Group 1b (G1b). RL, total root length; RA, total root area; RTD, root tissue density; TRL, tap root length; RFW, root fresh weight. The number after the underscore indicates the order of image acquisition.

The hierarchical cluster analysis separated the 40 spinach accessions into four major groups using a distance of 20, and Group 1 was further divided into two subgroups as Group 1a (G1a) and Group 1b (G1b) (Fig. 3). The sample root X-ray images of accessions under the five identified groups are shown in Fig. 3C.

Group 1 (G1) gathered together 18 accessions including 7 accessions in G1a and 11 accessions in G1b. Compared to other groups, the accessions in G1a had relatively lower RL, TRL, RA, RTD, MRG_TRL, and MRG_RL, and all tested traits (except the mean growth rate of tap root length between the second and third imaging periods (MRG_TRL23)) were below the average of all groups (Fig. 3B, Table 5). This suggested that the accessions in G1a had a small and shallow root system during the three vegetative growth stages. The RL, RA, and RTD of the accessions in G1b were also below the mean values of all groups as those in G1a, but their TRL and MRG_TRL were close to the mean values, suggesting that the accessions in G1b had a thinner tap root.

**Table 5:** Comparison of the normalized values of eight root traits and six shoot traits between the five groups identified in cluster analysis.

Trait	G1a	G1b	G2	G3	G4	Trait	G1a	G1b	G2	G3	G4
TRL_1	− 0.20	0.00	0.29	− 0.15	0.06	RFW	− 0.21	− 0.31	0.94	− 0.06	− 0.36
TRL_2	− 0.22	− 0.01	0.33	− 0.17	0.07	SRL	− 0.22	− 0.01	− 0.6	0.21	0.25
TRL_3	− 0.16	0.03	0.09	0.00	0.05	PB_1	− 0.11	− 0.05	0.12	0.03	0.01
RL_1	− 0.27	− 0.29	− 0.01	0.03	0.54	PB_2	− 0.13	− 0.08	0.24	− 0.02	0.00
RL_2	− 0.37	− 0.21	0.03	0.02	0.53	PB_3	− 0.12	− 0.05	0.17	− 0.03	0.04
RL_3	− 0.45	− 0.13	0.12	0.44	0.02	PH_1	− 0.18	− 0.04	0.20	− 0.03	0.05
RA_1	− 0.19	− 0.37	0.03	− 0.05	0.59	PH_2	− 0.16	− 0.02	0.16	− 0.02	0.04
RA_2	− 0.36	− 0.24	0.09	0.02	0.49	PH_3	− 0.1	− 0.03	0.34	− 0.1	− 0.11
RA_3	− 0.46	− 0.10	0.16	0.38	0.02	OA_1	− 0.33	− 0.18	0.47	0.01	0.02
RTD_1	− 0.23	− 0.23	− 0.04	0.05	0.44	OA_2	− 0.37	− 0.12	0.55	− 0.05	0.01
RTD_2	− 0.33	− 0.24	− 0.01	0.15	0.43	OA_3	− 0.33	0.01	0.45	− 0.09	− 0.03
RTD_3	− 0.42	− 0.15	0.09	0.49	− 0.01	OV_1	− 0.48	− 0.21	0.67	− 0.01	0.04
MRG_RL12	− 0.37	0.09	0.07	0.13	0.09	OV_2	− 0.5	− 0.11	0.70	− 0.09	0.00
MRG_RL13	− 0.39	− 0.19	0.29	0.5	− 0.2	OV_3	− 0.44	0.06	0.8	− 0.23	− 0.2
MRG_RL23	− 0.33	− 0.13	0.24	0.5	− 0.28	LL_1	− 0.12	− 0.08	0.13	0.03	0.04
MRG_TRL12	− 0.31	0.05	0.32	− 0.12	0.06	LL_2	− 0.16	− 0.06	0.16	0.03	0.04
MRG_TRL13	− 0.06	0	0	0.09	− 0.03	LL_3	− 0.21	0.01	0.19	− 0.02	0.04
MRG_TRL23	0.04	− 0.02	− 0.13	0.18	− 0.07	SFW_3	− 0.26	0.02	0.44	− 0.07	− 0.14

The normalized value was calculated as the quotient of the trait value in a group minus the mean value of all groups divided by the mean value. The number after the underscore indicates the order of image acquisition.

*RL* Total root length, *RA* Total root area, *RTD* Root tissue density, *TRL* Tap root length, *MRG_TRL* Mean growth rate of tap root length, *MRG_RL* Mean growth rate of total root length; The number after the underscore indicates the order of image acquisition.

The accessions in Group 2 (G2) had above-average TRL and the highest RFW of other groups, and their RL, RA, and RTD were close to mean values. The G2 root ideotype can be illustrated as a large, long/deep root system (Fig. 3B, Table 5). Additionally, the mean growth rate of total root length between the first and third (MRG_RL13), the second and third (MRG_RL23) imaging periods, and the mean growth rate of tap root length between the first and second imaging periods (MRG_TRL12) in G2 were above average, indicating both their tap root and lateral root developed more rapidly than other groups. For the accessions in Group 3 (G3), their TRL was close to average and the RL, RA, and RTD were above average at 43 DAT (Fig. 3B, Table 5). Moreover, the MRG_RL13 and MRG_RL23 in G3 were also greater than those in other groups, indicating the accessions in G3 had a strong root system at the later stage. Different with the group of G3, the root system of the accessions in Group 4 (G4) could be characterized as an early-strong root system. This group had relatively high values of RL, RA, and RTD at the first two stages, and their average TRL was above the mean of all groups at the three stages (Fig. 3B, Table 5).

The value of each shoot trait relative to the mean value was also analyzed (Table 5). The accessions in G1a with small and shallow root systems had relatively low values of all tested shoot traits (PB, PH, OA, OV, and LL). Their shoot traits were also below average at each stage. The accessions in G1b had similar shoot traits as G1a, but their OA_3 (OA at 4 DAT), OV_3 (OV at 43 DAT), and LL_3 (LL at 43 DAT) were higher than those in G1a, and even reached the mean values of all groups. The accessions in G3 and G4 had almost similar shoot traits. All their five shoot traits were near the mean levels at each stage, except the OV in the third period (OV_3), which was below the average. The relatively high values of five shoot traits (PB, PH, OA, OV, and LL) were found in the accessions of deep root group G2, with all these values being above the average.

The percentage of each group's leaf shape was also calculated. All the accessions in G2 had halberd-shaped leaves, while the percentage of halberd-shaped leaves in G1a, G1b, G3, and G4 was 42.86%, 36.36%, 36.36%, and 14.29%, respectively.

To further verify the results of cluster analysis and characterize the overall root performance of each accession, a mean MFV based on each germplasm accession for each root trait was calculated, and the accessions were ranked based on their mean MFV (Supplementary Table S5). The mean MFV ranged greatly from 0.11 to 0.62, with a lower mean MFV in the G1 group and a higher MFV in other groups. As expected, the accessions remained in a similar ranking with the results of the cluster analysis. All accessions in the G1a group had the lowest mean MFV, followed by the G1b group members, while the other three groups ranked higher. According to the mean MFV from tap root traits, the accessions in G2 and G4 also had a significantly greater TRL, consistent with the results from cluster analysis (Supplementary Table S5). Combining the results from two methods, spinach accessions in this study can be briefly classified into two categories: large (G2–G4) and small root (G1) types.

## Discussion

Variability in root traits is critical in attempts to genetically improve crop performance and response to changes in the production environment. Studying root phenomics is increasingly becoming a crop-breeding strategy, and efforts have been made to develop automated, high-throughput and reliable root phenotyping methods for crop breeding and research. However, most of the reported studies on spinach root phenotyping mostly focused on the traits identified at one stage but did not note how this would happen at other periods^24,26^. In the present study, a non-destructive RootViz FS system that could persistently track plant root growth under controlled conditions was used to quantify root system characteristics among 40 spinach accessions.

In this system, plants were grown in a low density foam medium in a controlled environment. The homogeneity of the medium and the controlled environment can help to minimize environmental error and maximize genetic differences between accessions. Simple traits such as total root length, tap root length, and maximal root width have been quickly and successfully obtained from high resolution images. In theory, the system can capture more root traits, such as root angle and diameter of intercepts at different depths, but due to the particularly thin lateral roots in spinach, these traits cannot be achieved accurately.

To analyze the relationship between roots and shoots, we simultaneously acquired shoot image data using another image acquisition system, Scanalyzer 3D. A previous study reported an image analysis pipeline that enables the simultaneous collection of shoot and root in carrots, which allows for rapid and low-cost acquisition of high-throughput phenotypic data for genetic studies^46^. The RootViz FS system used in this paper can theoretically enables the scanning of the root and shoot phenotypes at the same time, especially for spinach in the early periods, when the leaves are small and less shade each other. As the leaves became larger and shaded each other, larger plants could exceed the scanning field of view, affecting the results of the experiment, so the more effective system, Scanalyzer 3D, was still used. Later, an experiment to determine the effect of employing RootViz FS on shoot morphology acquisition can be conducted.

The 40 spinach materials selected in this study were derived from more than 200 spinach core germplasms with good representativeness and high genetic diversity. The coefficient of variation (CV) of shoot traits varied greatly, ranging from 0.15 (PB_1) to 0.91 (OV_3), suggesting a large variation in shoot traits among spinach genotypes. Compared to shoot traits, larger CV values between accessions were observed for most of the root traits at all stages examined. It is well known that root architecture traits like RL, RA, and RTD are crucial for the plant's response to water and nutrient acquisition^3^. Their larger variations among genotypes have been reported in other plants, such as wheat^11^, sesame^47^, and grapevine^48^. All these indicated great intrinsic diversity in spinach roots, which could allow the phenotypic screening for new cultivar breeding at a given place and time^49^.

Most importantly, MRG_RL had the largest CVs of all measured root traits, highlighting the importance of temporal root growth patterns in the analysis of phenotypic variability and genetic diversity in spinach. The dynamic patterns of root growth were also significantly reflected by the decreasing correlations with increasing sampling intervals and the relatively higher values, folds, and CVs of each trait than those among the early periods. This is possibly due to the rapid root growth during the later periods (30 to 43 DAT). The different response of the root system during spinach growth demonstrated the strong genotype-by-time interactions in spinach roots, which may support spinach performance across a wide range of growth conditions.

Nevertheless, considerable correlations of the same traits were found among different imaging times, such as RL, RA, and RTD. This implied that these root-related traits have developmental relevance. Similar results were obtained by Wang and coworkers, who reported the significant correlation between the traits studied among six vegetative stages in *Brassica napus* L., and suggested that root development is controlled by common genetic factors with prolonged effects^9^. The strong and positive correlations among RL, RA, and RTD were also in agreement with previous studies reported in wheat^50^, watermelon^13^, suggesting that these phenotypes can be used simultaneously for spinach root architecture trait improvement. Furthermore, most of the tested traits showed high stable heritability under the RootViz FS system, indicating that these traits can be selected for breeding prediction.

The strong positive correlation between root and shoot traits has been widely reported in previous studies, and certain root traits likely to improve nutrient acquisition, yield, or abiotic stress tolerance have been suggested for breeding programs, as mentioned before^1,51,52^. Interestingly, in this study, we found that the correlations between shoot and root traits in spinach depended on their developmental stages. In the early period, only TRL was weakly correlated with shoot traits (PH, LW, OA, and OV), suggesting that the tap root length may be more representative of the overall morphology of the early root system than the whole root size. However, root biomass-related traits, such as RFW, RL, RA, and RTD, strongly correlated with shoot biomass-related traits at the later period, indicating the more essential role of root size in the later period than root depth in root uptake of water and nutrients for plant aboveground development.

The distinct dynamics of tap roots and lateral roots in spinach may be a mechanism for the plant's own growth or adaptation to environmental cues^53^. During the seedling stage, resources were allocated to primary root growth to provide sufficient space for the initiation and establishment of the first-order lateral root^9^. Root depth as an important root trait in plant water and nutrient uptake has been widely reported for improved biomass production^43^. Therefore, tap root length may play a similar role as root depth in plant establishment here. As the plants continued to grow, the distribution of resources tended towards lateral root development, and the lateral roots were the main contributors to the root length in spinach. This allocation between lateral and primary root growth at different periods maximized the root uptake area, thereby improving biomass accumulation in spinach. This finding was partly supported by the performance of 40 accessions, which were divided into four groups. Among them, the G2 accessions had early deeper tap roots and stronger lateral roots at later periods, as well as higher root vigor (MRG_TRL and MRG_RL) at both early and later periods, so it is not surprising that they had the greatest biomass of root and shoot than other groups, demonstrating the importance of changes in root distribution. In contrast to G2, the accession in G1, with the lowest trait values related to tap root and lateral root, showed a lower biomass of both root and shoot. This is consistent with the previous findings that the deep and vigorous root system is ideal for achieving high yields in most field crops^18^.

For G3, though the accessions in G3 had higher RL, RA, and RTD at 43 DAT, their shoot biomass was lower than those in G2. Considering the great importance of lateral roots in foraging for nutrients due to their ability to increase the uptake area of a root system^54^, the significantly lower TRL in G3 at the early period may be the main reason for the lower shoot biomass at 43 DAT, which affected the initiation and distribution of the lateral roots, thus affecting the nutrient absorption function of the root system and the shoot biomass accumulation at the later period.

Different with G3, the accessions in G4 had relatively well-developed TRL and RL at the early period, but their lateral root-related traits at the later period were lower than in G2, resulting in a relative poor performance of the shoot and root compared with G2. In spite of this, G4 had a relatively better performance in the early period than other groups (except G2), which highlighted the crucial role of taproot development at the seedling stage. The differentiated root performance between the G3 and G4 groups after long-term growth indicated significant developmental stochasticity in plant roots. All these revealed large intrinsic temporal phenotypic variations in spinach roots, and their strong link to plant establishment should be noted. Based on these results, we proposed that early deep tap roots and later strong lateral roots can be selected as candidate traits for breeding spinach cultivars with improved resource acquisition. So, if crop yield is the main consideration for the spinach breeding target, the G2 root type may be a promising idotype for breeding, while the G4 root type with early below-ground vigor may be advantageous for quick harvesting of spinach. As for two other types of root systems, their detailed function under diverse environmental and nutritional stresses deserves to be investigated in the future.

We also found that the spinach root systems were somewhat related to their leaf shapes. The spinach with halberd-shaped leaves tended to have larger root systems than those with nearly orbicular-shaped leaves, and the accessions with strong root systems in G2 all had halberd-shaped leaves. Available morphological information indicated that wild spinach usually has a narrow, hastate, and smooth leaf shape^30,55^. So this seems to suggest that the halberd-leaved spinach was more evolutionarily primitive and better adapted to environmental changes, resulting in a more developed root system and more rapid growth of the above-ground parts. Further experiments are needed to verify it.

## Conclusion

This study evaluated the temporal patterns of root growth in a collection of spinach germplasm. The wide dynamic variability in root traits at three imaging times was found among spinach accessions. Though common genetic factors controlled root development with long-term effects, strong genotype-by-time interactions existed in spinach roots. The 40 spinach accessions were classified into four major groups with distinct tap root and lateral root growth patterns. Among them, germplasm with early deeper tap roots and later stronger lateral roots can be used as promising parental choices to improve spinach biomass accumulation.

### Supplementary Information

Supplementary Information.

## Data Availability

The datasets used and/or analyzed during the current study available from the corresponding author on reasonable request.
